# The experiences of patients with COVID-19 and their relatives from receiving professional home care nursing: a qualitative content analysis

**DOI:** 10.1186/s12912-024-02021-9

**Published:** 2024-05-27

**Authors:** Mina Shayestefar, Nayyereh Raiesdana, Monir Nobahar

**Affiliations:** 1 Student Research Committee, Semnan University of Medical Sciences, Semnan, Iran; 2 Nursing Care Research Center, Semnan University of Medical Sciences, Semnan, Iran; 3 Department of Nursing, Faculty of Nursing and Midwifery, Semnan University of Medical Sciences, Semnan, Iran; 4 Social Determinants of Health Research Center, Semnan University of Medical Sciences, Semnan, Iran

**Keywords:** COVID-19, Nursing, Home care, Qualitative study, Content analysis

## Abstract

**Background:**

To overcome of patients with COVID-19 over the capacity of hospitals and mild to moderate severity of the disease in most cases, the World Health Organization and the Centers for Disease Control and Prevention in the United States, recommend home care for these patients. Receiving care at home will face challenges that can be context-based, especially in crises like the Coronavirus pandemic. The present study aimed to describe the experiences of patients with COVID-19 and their relatives from receiving professional home care nursing.

**Methods:**

This study was conducted using a qualitative content analysis method. Nine participants with COVID-19 who were receiving home care nursing in Semnan participated in this study. The purposive sampling method was used. Sampling continued until no new categories appeared, meaning the category’s theoretical saturation. Deep and semi-structured interviews were used to collect data based on the research question. Data was analyzed using the conventional content analysis method using Graneheim and Lundman’s approach.

**Results:**

After analyzing the interviews and comparing codes based on similarities and differences, three main themes, 11 categories, and 30 subcategories were identified. The main themes included “The value of home care” (personalization of care, being economical, providing intellectual security, and reducing the concern of family), “Comprehensive care” (professional commitment, empathy, mastery in care, and patronage), and “Care challenges” (cultural barriers, inadequate services, and lack of information about costs and conditions).

**Conclusion:**

The patients with COVID-19 who received professional nursing care at home mentioned some challenges, such as the caregiver not being of the same sex as the patient, delay in receiving the service, the inadequacy of the centers, the limitation of the right to choose the care provider, and insufficient information about the cost of services received before receiving each care.

## Background

COVID-19 as a pandemic crisis put unprecedented pressure on healthcare services [1]. The experience of caring for patients with some infectious diseases such as SARS shows that the evaluation and care of these patients are costly and require a lot of facilities and human resources. In addition, providing care increases the risk of transmitting the disease to other patients, nurses, and medical staff [2]. Approximately 81% of COVID-19 patients show mild symptoms and can be recovered at home; therefore, quarantine and home care are some of the common treatments for this disease [3]. In early 2020, the World Health Organization and the United States Centers for Disease Control and Prevention recommended that people with COVID-19 receive care at home, emphasizing that most cases are mild or moderate [4].

Today, the increasing number of people needing home care services, especially the elderly and chronic disease patients, have taken nursing care from hospitals and nursing centers to home care [5]. Providing care at home allows patients to choose the care provider and the amount of nursing services according to their willingness and choice [6]. During the COVID-19 home quarantines, home care services may act as an essential auxiliary component of health care services, which reduces the burden on the formal health care system [7, 8].

Home care nursing services are essential to community-based integrated care systems to support people needing care in the community [9]. Nursing services at home have been significantly developed in the last twenty years to meet the population’s needs and adapt to the health system’s limitations [10]. Home care can maintain safe care during the pandemic due to COVID-19, with a low incidence of COVID-19, low hospitalization rate, and low mortality [11].

Preliminary regulations of home care services have been approved since 1999 in Iran and then revised in 2014 [12]. Although the movement toward professionalization in-home care has begun in Iran, there is still a long way to go [13].

Home care nurses provide a wide range of professional and supportive health services such as patient education, wound care and treatment [14], rehabilitation treatment, social work, and diet [15]. They are often the first to recognize and report the deterioration of the patient’s condition or the manifestation of dangerous symptoms [14].

Jacob et al. (2021) and De Mestral et al. (2019) have stated that nursing at home may help reduce emergency visits and re-hospitalization [16]. As Heyn et al. (2021) noted in their study, most of the concerns during the visits are related to primary nursing care and/or medication administration and the type of home visit (including providing basic needs, medication administration, and nursing procedures altogether or separately) may affect the expressed concerns of the elderly. However, it seems that the complexity of the health condition of the elderly has little effect on the expressed concerns [17].

The research findings related to home care nursing can provide primary data for the use and policy-making of home care nursing [15, 18]. COVID-19 has made a wide range of changes in all aspects of healthcare systems. Also, it reveals the gaps in providing healthcare, which should be reflected and may help improve home care services and cope with other situations. One of the best quality assessment methods in healthcare can be the receiver care’s viewpoints and experiences. Therefore, the present study was conducted to describe patients’ experiences with COVID-19 and their relatives from receiving professional home care nursing with a qualitative approach in Semnan, Iran.

## Materials and methods

### Qualitative approach

This study was conducted using conventional content analysis, according to the objective of this study.

### Research population

The research population consisted of all the patients with COVID-19 and their relatives in Semnan, Iran, who received nursing care at home and were willing to participate in this research.

### Participant selection

Inclusion criteria were: participants’ age of 18 years and above, the experience of receiving home care nursing at least once due to COVID-19 in 2022, absence of COVID-19 disease at the time of sampling, willingness to participate in the current study, ability to share their experience, and cognitive disorders. Also, exclusion criteria were sudden changes in the participant’s physical or mental status leading to the inability to communicate and the participant withdrawing from continuing cooperation for any reason. As stated in the research title, the participants were patients or their family members. Due to speech impairments and the death of some eligible participants, researchers had to interview the closest family member who was in connection with the home care center instead of the patients to gather important information in these situations.

The purposive sampling method was used in the present study. After seven interviews, new data did not emerge, and the following two interviews were conducted to ensure that no new data emerged. Data gathering was done from June 2022 to December 2022.

### Data collection

The data collection tool was in-depth and semi-structured interviews based on the research question. Also, during the interviews, field notes were taken. A total of ten interviews with nine participants were conducted (Table 1).

**Table 1:** Characteristics of the participants, relatives, and interviews

Number	Interviewee	Sex	Age (y)	Marital status	Job	Level of Education	Interview duration (min)	Place of the interview
1	Patient	Female	67	Widowed	Retired teacher	Diploma	36 (1st ) 12 (2nd )	Patient’s house
2	Patient’s son	Male	52	Married	Retired employee	Master	22	Park
3	Patient	Female	64	Married	Housewife	Primary	22	Nursing faculty interview room
4	Patient	Male	78	Married	Retired employee	Diploma	46	Patient’s house
5	Patient	Male	40	Married	Employee	PhD student	29	Nursing faculty interview room
6	Patient’s daughter	Female	50	Married	Housewife	Primary	26	Park
7	Patient’s son	Male	43	Widowed	Company manager	Bachelor	18	Participant’s workplace
8	Patient	Male	27	Single	Employee	Master	32	Nursing faculty interview room
9	Patient	Male	25	Single	Shopkeeper	Diploma	16	Park

After approving the research project and obtaining the ethics code (IR.SEMUMS.REC.1401.039), the first author (M.Sh.) went to all of the nursing centers that provide home care in Semnan City and received the list of patients with COVID-19. Then, an appointment was made with the eligible informants during a phone call, and the research objectives were explained.

The location of the interviews was coordinated by the suggestion of the participants and in a place with the least disturbing factors. Participants completed the informed consent form during the meeting.

All interviews were conducted by a first author (female faculty member, Ph.D. candidate in nursing). With the participants’ permission, all the interviews were recorded by the MP3 player without mentioning any names during the interview. They were informed that their information is confidential and anonymous and will be used in the research process by assigning a number. The audio file was transcribed verbatim immediately after each interview, and the meaning units were extracted by the interviewer (first author, Ph.D. candidate in nursing) and verified by the research team. The average duration of the interviews was 28.77 ± 11.48 (min 16, max 48) minutes.

Initially, a general question was asked based on the research aim: “*Please describe your experience of receiving nursing care at home during the COVID-19*”. In addition, during the interview, some guiding questions were added, including “*Please describe your feelings about receiving nursing care at home during the COVID-19*” and “*How would you describe receiving nursing care at home during this disease*?” so that the participants explained their experiences more about receiving home care nursing. Also, based on the participant’s answers and the interview progress, probing questions such as “*What do you mean*?”, “*If you can explain more?*” were asked. The path of the following interviews was determined through data collection, simultaneous analysis, and the formation of categories. After several interviews, other questions were added based on the research progress. The participants’ non-verbal messages, such as tone of voice, silence, emphasis, and sighing, also were recorded.

### Data analysis

For data analysis, the first author (M.Sh.) listened to each interview and read the transcription several times better to understand the feelings and experiences of the participants. The approach used for data analysis was Graneheim and Lundman’s (2004) conventional content analysis. According to this approach, the text of the interviews was transcribed verbatim and divided into meaning units (by M.Sh.). Then, meaning units were coded by two researchers separately (M.Sh. and N.R.). According to the participants’ experiences, evident and hidden concepts were determined from sentences, paragraphs, or words, and then coding and summarization were done. Based on the continuous comparison of similarities, differences, and appropriateness, the codes that indicate a single subject were placed in one subcategory, and the subcategories were merged to form the categories. Ambiguous points that need attention, in addition to being reviewed by the participants, were also explored in subsequent interviews (the first participant interviews). In such a way, the ambiguities were resolved, and the location of the codes in each category was fully specified. At the interpretation level, the central concept of each category was determined, and the primary and abstract concepts or themes were extracted [19].

Data analysis was done in the same way and simultaneously with data collection. Four criteria developed by Lincoln and Guba- credibility, transferability, dependability, and confirmability- were used to define the quality criteria of qualitative studies.

In the present study, prolonged engagement with the data and spending enough time to collect and analyze the data, data triangulation, member check, and peer check were used to verify the credibility of the data.

For transferability, the conditions of using the results in other contexts were provided for the readers by carrying out deep, analytical, and rich descriptions of the context and characteristics of the participants, describing the study context and precise description of limitations.

For dependability, data and documents were carefully reviewed by an external reviewer. The dependability of data is in similar time and conditions and is equivalent to reliability in quantitative research. In this study, the dependability of the data was determined in such a way that the data obtained in the interview also emerged in field notes. In this study, to achieve the standard of confirmability, all the stages of conducting the research, especially the stages of data analysis, were recorded in a detailed and comprehensive manner so that if another researcher who wants to continue research in this field, follows the work process based on the documents quickly. In addition, all interviews were reviewed after transcription in face-to-face meetings with all research team members, and their corrective comments were applied.

## Results

After analyzing the interviews and comparing codes based on similarities and differences, three main themes, 11 categories, and 30 subcategories were identified (Table 2).

**Table 2:** Themes, categories and subcategories

Main theme	Category	Subcategory
The value of home care	Personalization of care	Being in a personal environment
		Availability of personal belongings
		Being with familiar persons
	Being economical	Reducing costs
		Saving time
		Coordination with medical centers is a necessity
	Providing intellectual security	Reducing the fear of hospitalization
		Reducing the mental load of disease-worsening
	Reducing the concern of family	No need to move the patient
		Easiness of availability of the required drugs
		Removing pressure care from family members
Comprehensive care	Professional commitment	Punctuality
		Availability
		Responsibility
		Compliance with health principles
	Empathy	Understanding the patient’s condition
		Being good-humored
		Effective communication
	Mastery in care	Clinical skill in meeting care needs
		Knowledge in providing care
	Patronage	Providing the necessary guidance
		Answering the patient’s questions
		Strengthening the morale
Care challenges	Cultural barriers	Opposite sex of patient and caregiver
		Accepting a stranger at home
	Inadequate services	Inability to receive long-term care
		Delay in receiving care
		A limited number of service delivery centers
	Lack of information about costs and conditions	Inadequate information on fees
		Lack of information about covered services

### The value of home care

The theme “value of home care” refers to the preference of home care from the patient’s point of view. In this theme, an implicit comparison was often made between receiving care at home and in the hospital. This theme includes four categories: “Personalization of care,” “Being economical,” “Providing intellectual security,” and “Reducing the concern of family.”

### Personalization of care

This category refers to the sense of ownership over one’s surroundings and the feeling of belonging and confidence that comes with it. This category includes three subcategories: “Being in the personal environment,” “Availability of personal belongings,” and “Being with familiar persons.”

#### Being in a personal environment

This subcategory refers to the patients’ interest and desire to receive care in a familiar climate; indeed, it means the patient’s home, which leads to a sense of security in the patient.

“It’s that feeling of security that comes from knowing you can care for yourself in your environment. For example, your home is a place you have control over 24 hours (making a circle with both hands). You can do whatever you want there, but it’s not like that in a hospital” (P8).

#### Availability of personal belonging

This subcategory refers to the feeling of independence over personal belongings and access to personal amenities such as the patient’s favorite food; also, the accessibility to personalized sanitary equipment was an important factor for them.

*“But I was at home because I was alone, and it was just me, and I was comfortable with myself. I took care of myself in terms of food and everything”* (P1).

#### Being with familiar persons

This subcategory refers to the patient’s preference for receiving care from a familiar and consistent nursing provider. This can provide care with more focus and exclusivity, allowing for more family involvement and presence.

*“Taking care at home is great. It’s better inside the house because one person’s focus, attention, and care are higher. They say they do their job better than being in the hospital with 10 or 20 patients or even a single room. Well, it’s different. They rush things in the hospital, but at home, they provide better care, focus, and attention to detail, and they do their job better… But having a doctor or nurse come to your home to care for you is even better since their focus is higher, and they do your job with greater accuracy and better care, which is much better”* (P9).

*“My father is also stressed, and it would be better if we were not around him in a crowded space, especially my sister, who gets extremely stressed. Plus, the COVID-19 section was there, which added to his stress. We returned home and told him we would care for him and see what happened next. We were sure he was stressed there, and being around someone stressed was not good for us. Being there for him emotionally makes a big difference”* (P7).

### Being economical

This category refers to the patients’ mental consideration of cost-effectiveness when receiving care, which includes three subcategories: “Reducing costs,” “Saving time,” and “Coordination with medical centers in necessity.”

#### Reducing costs

The subcategory of “reducing costs” does not necessarily mean less spending on home care but rather refers to the value of the cost spent versus the care received.

*“To be honest, I wasn’t focused on the cost and how it was or what it was. I didn’t pay too much attention to it. But I feel like it was worth it. Let me put it this way”* (P5).

#### Saving time

This subcategory refers to reducing the time commitment for the patient and family (such as waiting time for appointments) in receiving care compared to care received outside the home.

*“When you go to a clinic, you have to wait in line and deal with the crowd, but it’s not like that at home. It’s much better”* (P5).

#### Coordination with medical centers in necessity

The following subcategory indicates providing telephone consultation for home care nursing in various fields with relevant individuals, which can accelerate the treatment process.

*“In terms of food, Dr. … said that she is a professor of traditional medicine at the university, and they gave me her phone number. She provided consultation over the phone on what to eat and what to do”* (P1).

### Providing intellectual security

Ensuring mental security refers to peace of mind and a sense of safety for the patient, which includes two subcategories: “Reducing the fear of hospitalization” and “Reducing the mental load of disease worsening.”

#### Reducing the fear of hospitalization

This subcategory refers to reducing the fear of illness, which involves the fear of hospitalization in medical centers, and home care can create a sense of comfort and security for the patient.

*“From the perspective of how much worse pollution is in a hospital environment, people are scared because COVID patients come and back… This fear is on the one hand, and the other hand, your home environment feels safer for this reason”* (P8).

#### Reducing the mental load of disease worsening

This subcategory has been developed based on reducing the mental pressure caused by hospitalization with COVID-19. However, when it becomes possible to provide care for the patient at home, their mental perception of the severity of the illness decreases.

*“When they say that taking care at home helps the patient breathe a sigh of relief, for example, they say, ‘Well, it’s not that severe that they have to hospitalize me.’ Because it always comes to their mind that whenever a doctor says to be hospitalized, people start saying, ‘Oh, what’s happening?’ and so on… and they convince themselves that there is nothing wrong, and they might get better in, say, five days a week”* (P8).

### Reducing the concern of family

The culture of excessive communication in Iranian families often leads to the involvement of family members in problems or even the illness of one of them. Home care, based on its features, can reduce the excessive participation and concern of the patient’s relatives. This category consists of three subcategories: “No need to move the patient,” “Easiness of availability to the required drugs,” and “Removing pressure care from family members.”

#### No need to move the patient

This subcategory refers to patients not needing to be transferred to healthcare centers for treatment and care. This situation is particularly more noticeable in elderly patients and those with a significant reduction in mobility.

*“Taking care at home is very effective, and my father was more comfortable. Every day, despite his old age, he didn’t have to struggle to get in and out of the car and travel. Not only was he a patient, but also without being sick, it is hard for him at his age”* (P2).

#### Easiness of availability to the required drugs

This subcategory refers to the purchase of medications from pharmacies which were near the patients’ homes, as well as the access to the medicines by home care nurses who have more information about different routes of drug purchase, especially in times of drugs shortages during the COVID-19 crisis in Iran.

*“As the saying goes, that person who comes buys and brings all these medicines, and the patient inside the house does nothing ………. There were some foreign vitamins. Mr. … himself didn’t take them, my sister went to one or two pharmacies and couldn’t get them, I don’t know where Mr. … got them from now”* (P9).

#### Removing pressure care from family members

Another subcategory of the essential patient experiences regarding reducing family concern is “relieving the caregiving pressure on family members”. This means that family members are less anxious and worried about caring for the patient by facilitating the caregiving process.

*“During the coronavirus, I got sick and was hospitalized. My two sons were very involved in caring for me because I needed a lot of help. Then the doctor called my sons and said you annoyed a lot in this way; take your father home and take care of him there. It’s perfect, especially now that the sons are too busy in their own lives.”* (P4).

### Comprehensive care

Comprehensive care refers to professional nursing care based on ethical principles. This theme includes four categories: “Professional commitment,” “Empathy,” “Mastery in care,” and “Patronage.”

### Professional commitment

The “Professional commitment” category refers to some professional nursing principles that are more prominent in caring for COVID-19 patients at home. It includes four subcategories: “Punctuality,” “Availability,” “Responsibility,” and “Compliance with health principles.”

#### Punctuality

Many participants mentioned time management is an essential professional commitment principle, as it leads to gaining the patient’s trust and accelerating the treatment process.

*“He used to come regularly on time”* (P1).

#### Availability

Patients who receive care at home must feel confident that the nurse can answer any questions or address any problems.

*“Most of the time, if something happens, I call him, and if I can’t go to his office because of my job, I tell him my problem over the phone, and then he tells me what medication to take and what to do. If I need an injection or something, I take it and go to his office. I am delighted with him for the treatment”* (P9).

#### Responsibility

Feeling responsible for providing nursing care was another expectation that patients had from their nurses.

*“Even Mr. …. himself wouldn’t come; he would call and ask about our condition for a while. He would check how I was doing, my situation, and whether I had improved or improved. This is very important”* (P9).

#### Compliance with health principles

During the COVID-19 pandemic, observing health principles was crucial for recipients of health care services. These health principles included wearing a mask, maintaining social distancing, washing hands, and using sanitizers.

*“They were wearing special COVID clothes, masks, gloves, and hats when they came in”* (P1).

### Empathy

Empathy is the ability to understand the patient’s feelings and experiences and for the nurse to look at the situation that COVID-19 has created for the patient from the patient’s perspective. In this study, empathy refers to “Understanding the patient’s condition,” “Being good-humored,” and “Effective communication.”

#### Understanding the patient’s condition

The most fundamental concept of empathy is “Understanding the patient’s condition.” The patient feels they have received care for their physical and emotional well-being.

*“Then, when he came, it was like he was doing this with all of his heart and soul*” (P4).

*“He used to work very lovingly when he came to our house.”* (P1).

#### Being good-humored

Another important subcategory of empathy is “Being good-humored.” Cheerfulness in home care nursing can lead to the continuity of care.

*“If I want to recommend home care to others, it all comes from the behavior and ethics of that nurse”* (P9).

*He used to come on time, was very gentlemanly, and had obvious eyes.* (P3)

#### Effective communication

The expectation that patients have from nurses is to have close and appropriate communication. Effective communication makes the patient feel safe and calm.

*“But on the other hand, he would ask about my well-being nicely. He would say, ‘How are you feeling now? What’s your condition like? Are you stable? Is your blood pressure okay? Do you feel nauseous? Do you have a fever?’ Everyone has their personality, so it all depends on the nurse who comes to take care of you”* (P9).

### Mastery in care

Mastery of clinical nursing skills brings patients trust and peace of mind, which can be based on professional nursing knowledge and practice. Mastery in care in the present study consists of “Clinical skill in meeting care needs” and “Knowledge in providing care.”

#### Clinical skill in meeting care needs

A patient’s expectation in the first stage of a nurse is to meet their care needs and master their profession.

*“I knew they were proficient in their work, and when they are proficient, I also feel calm.”* (P4).

Knowledge in providing care means having the necessary understanding, skills, and expertise to provide effective and efficient care to individuals who require it. This knowledge can come from various sources, including formal education, training, and experience in healthcare or social services.

*“Their high level of knowledge helped me greatly with my treatment since I became ill”* (P9).

### Patronage

During the COVID-19 pandemic, due to the unique conditions created (such as the need for home quarantine, controlling anxiety caused by family illness, unfamiliarity with the dimensions of the disease, etc.), the supporter and advisor role of home care nurses is highlighted. Support includes three subcategories: “Providing necessary guidance,” “Answering the patient’s questions,” and “Strengthening the morale.”

#### Providing the necessary guidance

Patients expect their nurses to be able to guide them in various aspects of their illness and demonstrate their expertise in different areas of the disease. In some cases, home care nurses would also seek guidance from qualified individuals if necessary.

*“They would order us to wear masks, wash our hands thoroughly, and clean our clothes when we arrived. Regarding eating food in the same place, they would recommend these things to us and emphasize the health care precautions in this regard”* (P2).

#### Answering the patient’s questions

The role of nursing counseling, feeling responsible for patients’ questions, and answering them were also among patients’ expectations.

*“He would answer the questions I asked him”* (P4).

#### Strengthening morale

*Another subcategory of “Patronage”* was obtained. Nurses can improve patients’ morale through their knowledge and experience so that they feel better emotionally. As mentioned below:

*“In my opinion, these centers that provide services could be much more effective if they could work with them on their mental well-being and provide counseling. Unfortunately, because my father was alone and had no one else, he was very effective in his morale and had an impact on his recovery when he talked to him”* (P2).

### Care challenges

The challenges of caring for COVID-19 patients include mentioning the problems patients face in receiving care at home, which has much to do with the community’s behavior and the patient’s individual and informational literacy. Three subcategories include “Cultural barriers,” “Inadequate services,” and “Lack of information about costs and conditions”.

### Cultural barriers

Due to the religious and cultural norms of society, the presence of a nurse of the opposite gender in the patient’s home is one of the challenges for patients. “Opposite sex of patient and caregiver” and “Accepting a stranger at home” are subcategories of cultural barriers.

#### Opposite sex of patient and caregiver

There is a sense of comfort in receiving care from a same-gender caregiver in the culture of many Iranian society members.

*“Since my mother was older, she was a bit uncomfortable. Because of her age, she was more restricted and did not allow herself to be seen. But anyway, she was sick, and we had no choice but to accept that injections had to be done… I saw that she was in a lot of pain during the injections, so I called Mr.… and asked if it would be possible that your wife, who is a home care nurse too, do my mother the injection”* (P5).

#### Accepting a stranger at home

The presence of confidence in receiving the entry of nurses into the house and the lack of organizational introduction strategies for them are among the challenges mentioned in patients’ experiences.

*“Because the number is low and you can’t be sure to find good ones, and there is also the issue of trust; they will come to our house”* (P2).

### Inadequate services

Unmet societal expectations and the mismatch between nursing centers and their evolving needs lead to the emergence of service delivery challenges at home, which participants have repeatedly mentioned. Inadequate service delivery includes three subcategories: “Inability to receive long-term care,” “Delay in receiving care,” and “Limited number of service delivery centers.”

#### Inability to receive long-term care

According to the participants, the lack of approved health system care centers that provide long-term care is felt.

*“If someone wants a caregiver to come to their house and care for them 24/7, they won’t find such a person at all”* (P7).

#### Delay in receiving care

Another challenge identified by the participants was that sometimes there seemed to be an excessive delay in receiving further care, which exceeded the patient’s or their family’s expectations.

*“You need an expert in treatment factors, for example, to take care of you 24/7 and check on you, but you don’t have this at home, and maybe it only happens once. For example, they come once, do what needs to be done, and then they may be unable to return”* (P8).

#### Limited number of service delivery centers

The subcategory “Limited number of service delivery centers” clearly indicates the lack of structural and human resources in the home care field that align with the community’s needs.

*“There are families who give a good amount of money despite financial difficulties, but there are also families who don’t care about the money aspect, but would not hire someone they don’t trust”* (P2).

### Lack of information about costs and conditions

Many participants expressed concerns about the lack of transparency regarding the types of nursing services covered by primary and complementary insurance and their costs. The category of “Lack of initial awareness of costs and conditions” also refers to “Inadequate information on pays fees” and “Lack of information about covered services.”

#### Inadequate information on pay fees

According to the participants’ experiences, ambiguity in the process and amount of payment is due to the lack of patient awareness by nursing centers before starting care services and insufficient knowledge of service tariffs at the community level.

*“However, it would be great if there was a tariff or something on a website for this system that people could look at. They say they will deposit it now; of course, the amount is not much, but having tariffs is not bad”* (P7).

#### Lack of information about covered services

Another subcategory of “Lack of information about costs and conditions” was “Lack of information about covered services.” Due to the unknown aspects of some dimensions of home care nursing in Iran, insurance companies have not provided good transparency, which was felt in patients’ experiences.

*“I didn’t understand what happened with its costs and supplements. They didn’t give me any supplementary insurance”* (P5).

## Discussion

The findings include three main themes, 11 categories, and 30 subcategories were identified. The main themes included “The value of home care” (personalization of care, being economical, providing intellectual security, and reducing the concern of family), “Comprehensive care” (professional commitment, empathy, mastery in care, and patronage), and “Care challenges” (cultural barriers, inadequate services, and lack of information about costs and conditions). The present study is unique because it explains the home care receiver’s experiences during COVID-19, a critical situation.

One of the subcategories obtained in the present study was “Knowledge in providing care,” which led to a sense of confidence among the participants. Given the importance of the knowledge foundation of a profession, this value can also be extracted from the participants’ statements. Aune and Struksnes also concluded from their study, based on the experiences of home care nurses, that nurses work with complex challenges in home care. Still, the criteria for success depend on the knowledge of patients and nurses, organizational aspects, and collaboration [20].

“Responsibility” is an essential aspect of “Comprehensive care,” based on the experiences of participants, which reflects the “Professional commitment” of home care nurses. In the study by Fatemi et al., attention was paid to the fundamental values of the main categories obtained from their understanding of home care nurses’ professionalism, which included the subcategory of adherence to laws and responsibility [21]. In both studies, responsibility is an essential part of the nursing profession.

In the present study, one of the subcategories of “Comprehensive care” was “Empathy,” which consisted of three subcategories: “Understanding the patient’s condition,” “Being good-humored,” and “Effective communication.” One of the skills of a nurse, especially in-home care, is to establish appropriate communication by ethical and professional principles. As Hemberg and Bergdahl have also pointed out in their study on palliative care at home, nurses can balance their actions in the moment and change their nursing actions according to the patient’s wishes through sensitivity and ethical perception, and by creating participation in their approach [22].

“Mastery in care” includes two subcategories: “Clinical skill in meeting care needs” and “Knowledge in providing care,” which were emphasized by most participants. Along with the new conditions created by COVID-19 in providing care, nurses also had to adapt to knowledge and clinical performance. In a qualitative study, Jia and colleagues extracted specialized nursing skills as a primary data category. They stated that to cope with the ethical challenges arising from nursing COVID-19 patients, nurses must gain knowledge and nursing skills related to COVID-19, which can improve their clinical performance in infectious cases [23].

Another important subcategory extracted from this study was “Strengthening morale,” obtained from the main category of “Comprehensive care.” This subcategory indicates the nurse’s role as a counselor, especially in crises such as COVID-19. Galehdar and colleagues have reported similar findings in the study. In their study, the category of the need for psychological counseling for patients indicates that COVID-19 patients even require psychological counseling before the need for nursing care. The study of Galehdar et al. has the same question through the nurses’ experiences. The finding revealed three main categories: care erosion, nursing professional growth, and necessities. They emphasized the emotional challenges of home care nurses during the COVID-19 including bad feelings of inefficiency, stress, excessive physical fatigue, the dilemma between care delivery, falling apart from their families, and the fear of infecting them upon returning home, which can affect the quality of patient care [24].

The subcategory of “Inadequate services” with subcategories of “Inability to receive long-term care,” “Delay in receiving care,” and “A limited number of service delivery centers” can stem from inadequate planning and support at higher levels of management and policy-making in nursing, which can pose challenges in care. As Joo and Liu pointed out in their systematic review study on barriers to nursing care for COVID-19 patients, insufficient support for nurses from hospitals and the healthcare system can contribute to this subcategory [25]. Also, Akbarbegloo et al.’s study about psychological care experiences of COVID-19 patients in the home revealed that these patients experienced the non-response of the treatment team and concerns about the persistent condition of the disease [26].

In the present study, two subcategories of “Cultural barriers” and “Inadequate services” were obtained from the main category of “Nursing challenges,” indicating the significant role of cultural and religious issues on various dimensions of Iranian society, as well as some managerial deficiencies and the need for updating service delivery structures from the participants’ experiences. Similarly, Heydari et al. identified cultural dimensions and insufficient infrastructure as two main categories obtained from the experiences of participants in their study on barriers to home care services in Iran [27]. One of the nursing challenges during COVID-19 was the cultural aspects of Iranian society are so related to the Islamic religion, which creates limitations to taking home care by an opposite-sex nurse. So, localization based on these differences needs the top manager facilitator role for adequate home care services. Using the evidence-based method, the present study describes these people’s experiences and reveals the need to expand these services in Iran.

One of the findings of the present study was the “Inability to receive long-term care” and “Delay in receiving care,” both of which are related to “Inadequate services” in this area of the nursing profession in Iran. Human resource issues are among the subcategories obtained in Lotfi Fatemi et al.’s study on home care in Iran. They stated that home care managers believe home care is not permanent in Iran for various reasons, and retaining nurses’ interests in this field is complicated. Therefore, recruiting and retaining nurses for the long term is a significant challenge [21].

Considering the advances in designing systems and medical information record systems, the use of technology in identifying and recording patient information and home care nurses should be approved and qualified. It should replace paper-based records and traditional methods. Plans have also been proposed to add home care nurse information to the EHR [28].

The findings of this research can help to understand the opinions and expectations of the beneficiaries of nursing care at home in the religious and Iranian cultural context. Also, these findings can help develop the infrastructure and solve the challenges of home care by presenting the participants’ experiences.

### Study limitations

This is the first study on the experiences of COVID-19 patients receiving home care in Semnan, Iran. Some other aspects of these patients’ experiences with different contexts are unexplored and need further studies. To increase the credibility of the data, efforts were made to interview participants who received care from other nurses and had different economic, cultural, and social backgrounds. Additionally, some patients were elderly and unable to speak or were deceased at the time of the study, and as a result, interviews were conducted with their closest relatives. Since some of the recipients of nursing care at home were not able to express their experiences accurately due to reasons such as speech impairments or old age, or some of them had died, one of their close family members was involved in the research to express their experiences.

## Conclusion

This study revealed that patients with COVID-19 who received professional nursing care at home had emphasized positive aspects and the comprehensiveness of nursing care provided at home. But also, they mentioned some challenges, such as the caregiver not being of the same sex as the patient, delay in receiving the service, the inadequacy of the centers, the limitation of the right to choose the care provider, and insufficient information about the cost of services received before receiving each care.

## Data Availability

The data that support the findings of this study are available from the corresponding upon reasonable request.

## Abbreviations

COVID: Corona Virus Disease
HER: Electronic Health Record
